# Survival of glucose phosphate isomerase null somatic cells and germ cells in adult mouse chimaeras

**DOI:** 10.1242/bio.017111

**Published:** 2016-04-21

**Authors:** Margaret A. Keighren, Jean H. Flockhart, John D. West

**Affiliations:** Genes and Development Group, Centre for Integrative Physiology, Clinical Sciences, University of Edinburgh Medical School, Hugh Robson Building, George Square, Edinburgh EH8 9XD, UK

**Keywords:** Chimaera, Chimera, Glucose phosphate isomerase, Glycolysis, Oocyte, Spermatozoa

## Abstract

The mouse *Gpi1* gene encodes the glycolytic enzyme glucose phosphate isomerase. Homozygous *Gpi1^−/−^* null mouse embryos die but a previous study showed that some homozygous *Gpi1^−/−^* null cells survived when combined with wild-type cells in fetal chimaeras. One adult female *Gpi1^−/−^*↔*Gpi1^c/c^* chimaera with functional *Gpi1^−/−^* null oocytes was also identified in a preliminary study. The aims were to characterise the survival of *Gpi1^−/−^* null cells in adult *Gpi1^−/−^*↔*Gpi1^c/c^* chimaeras and determine if *Gpi1^−/−^* null germ cells are functional. Analysis of adult *Gpi1^−/−^*↔*Gpi1^c/c^* chimaeras with pigment and a reiterated transgenic lineage marker showed that low numbers of homozygous *Gpi1^−/−^* null cells could survive in many tissues of adult chimaeras, including oocytes. Breeding experiments confirmed that *Gpi1^−/−^* null oocytes in one female *Gpi1^−/−^*↔*Gpi1^c/c^* chimaera were functional and provided preliminary evidence that one male putative *Gpi1^−/−^*↔*Gpi1^c/c^* chimaera produced functional spermatozoa from homozygous *Gpi1^−/−^* null germ cells. Although the male chimaera was almost certainly *Gpi1^−/−^*↔*Gpi1^c/c^*, this part of the study is considered preliminary because only blood was typed for GPI. *Gpi1^−/−^* null germ cells should survive in a chimaeric testis if they are supported by wild-type Sertoli cells. It is also feasible that spermatozoa could bypass a block at GPI, but not blocks at some later steps in glycolysis, by using fructose, rather than glucose, as the substrate for glycolysis. Although chimaera analysis proved inefficient for studying the fate of *Gpi1^−/−^* null germ cells, it successfully identified functional *Gpi1^−/−^* null oocytes and revealed that some *Gpi1^−/−^* null cells could survive in many adult tissues.

## Abstract (precis)

**Summary:** Some *Gpi1^−/−^* somatic cells, lacking the glycolytic enzyme GPI, survived in mouse chimaeras. Breeding experiments showed that some GPI-null oocytes and spermatozoa were viable and functional.

## INTRODUCTION

The dimeric glycolytic enzyme, glucose phosphate isomerase (GPI; E.C. 5.3.1.9), also known as glucose-6-phosphate isomerase, phosphoglucose isomerase or phosphohexose isomerase, catalyses the conversion of glucose-6-phosphate to fructose-6-phosphate at the second step in glycolysis and the reverse reaction during gluconeogenesis in some cell types. In mice, GPI is encoded by a single, ubiquitously expressed, autosomal gene, *Gpi1,* on chromosome 7.

Evidence has accumulated that several different non-enzymatic, paracrine and autocrine functions are also mediated by one or more forms of secreted, extracellular monomeric GPI, which bind to cell membrane receptors (reviewed by Henderson and Martin, 2014; Jeffery, 1999; Kim and Dang, 2005). These proteins have more restricted tissue distributions than the ubiquitous dimeric GPI enzyme and may be truncated forms of the GPI monomer with different quaternary structures (Baumann and Brand, 1988; Mizrachi, 1989; Repiso et al., 2008). The term ‘protein moonlighting’ has been coined to describe proteins, such as GPI, that can perform multiple functions (Jeffery, 1999) and databases of these proteins are now available (see Henderson and Martin, 2014).

Thus, in addition to enzymatic GPI, the *Gpi1* gene encodes the neurotrophic factor, neuroleukin, NK (Chaput et al., 1988; Faik et al., 1988; Mizrachi, 1989), the autocrine motility factor, AMF (Niinaka et al., 1998; Sun et al., 1999) and the maturation factor, MF, which is capable of mediating differentiation of leukaemia cells to monocytes (Xu et al., 1996). GPI/AMF is secreted by tumour cells, protects cells from endoplasmic reticulum stress (ER stress) and apoptosis, and promotes cell motility, epithelial to mesenchyme transition and invasion and metastasis of tumour cells (Fu et al., 2011; Funasaka et al., 2009; Kim and Dang, 2005). In addition, GPI has been identified as a specific inhibitor of myofibril-bound serine proteinase in fish (Cao et al., 2000; Han et al., 2014). Finally, to confirm its remarkable ‘protein moonlighting’ multifunctional behaviour, GPI has been shown to promote embryo implantation in ferrets (Schulz and Bahr, 2003, 2004).

The mouse *Gpi1^a-m1H^* null mutation (hereafter abbreviated to *Gpi1^−^*) is thought to alter the protein structure around the active site of the enzyme (Pearce et al., 1995). Heterozygous *Gpi1^+/−^* mice are viable and fertile but *Gpi1^−/−^* homozygotes fail to complete gastrulation (Kelly and West, 1996). This is likely to be solely due to the glycolytic deficiency rather than, for example, impaired epithelial to mesenchyme transition during gastrulation caused by an abnormal GPI/AMF monomer. This is because monomers have no GPI enzymatic activity and mutants that eliminate human GPI enzymatic activity do not affect the other functions of the GPI monomer (Tsutsumi et al., 2003). Mouse GPI produces a testis-specific, minor isozyme (Buehr and McLaren, 1981), which appears to be a splice variant, lacking exons 5 and 6 (Vemuganti et al., 2010). However, the *Gpi1^−^* null mutation that we used produces no enzymatic activity in mouse testes (Peters and Ball, 1990). Thus, the second step of glycolysis will be blocked in male germ cells and spermatozoa as well as other cell types.

Although homozygous *Gpi1^−/−^* null mouse embryos die, the homozygous *Gpi1^−/−^* null genotype is not necessarily cell-lethal. For example, homozygous *Gpi1^−/−^* null cells were able to survive at low levels in fetal *Gpi1^−/−^↔Gpi1^c/c^* mouse chimaeras but they contributed better to the placenta and extraembryonic endoderm than to fetal tissues (Kelly and West, 2002a). Similarly, tumours of GPI-deficient, Chinese hamster cells were able to grow slowly in nude mice (Pouysségur et al., 1980). Characterising to what extent cells and gametes with embryo-lethal enzyme defects, such as the homozygous *Gpi1^−/−^* genotype, can survive in mouse chimaeras may help identify how such mutant and wild-type cells interact and also help identify alternative pathways and redundancy in metabolic networks. Although the survival of *Gpi1^−/−^* null cells has been characterised for fetal mouse chimaeras (Kelly and West, 2002a), there is only one preliminary report of an adult *Gpi1^−/−^*↔*Gpi1^c/c^* chimaera (Kelly and West, 2002b). This was a fertile female that produced oocytes, derived from *Gpi1^−/−^* null germ cells, which were capable of being fertilised and developing into fertile heterozygous *Gpi1^c/−^* offspring. However, this chimaera died so the contribution of *Gpi1^−/−^* null cells to adult tissues was not investigated in detail and no adult male *Gpi1^−/−^↔Gpi1^c/c^* chimaeras were produced. It remains unclear whether *Gpi1^−/−^* null cells can survive in many adult tissues and if *Gpi1^−/−^* null gametes can produce functional spermatozoa that are able to compete with wild-type spermatozoa to fertilise oocytes.

The aims of the current study were (i) to characterise the extent of survival of homozygous *Gpi1^−/−^* null cells in adult mouse chimaeras, (ii) to extend the previous preliminary study to evaluate whether female *Gpi1^−/−^↔Gpi1^c/c^* chimaeras can produce offspring from GPI-null oocytes and (iii) to determine whether male *Gpi1^−/−^↔Gpi1^c/c^* chimaeras can sire offspring from GPI-null spermatozoa derived from homozygous *Gpi1^−/−^* null germ cells.

## RESULTS

### Identification of adult *Gpi1^−/−^↔Gpi1^c/c^* chimaeras

**Fig. 1.**
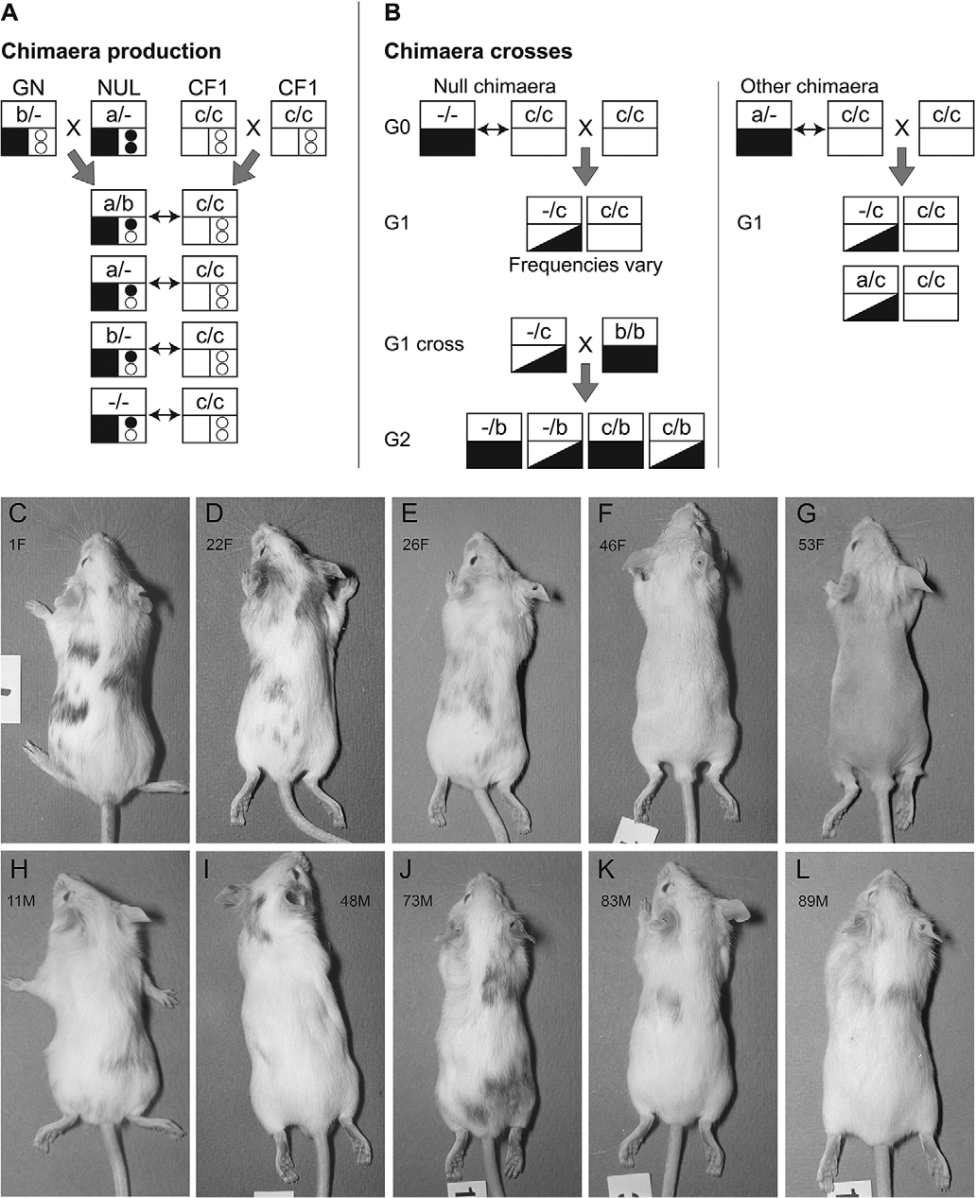
**Chimaera production, test breeding and coat colours.** (A) Diagram of genetic crosses used to produce chimaeras of four genotype combinations. GN mice were crossed to NUL mice to produce embryos of four pigmented, *Gpi1* genotypes, which were aggregated to albino, *Gpi1^c/c^* embryos from CF_1_×CF_1_ crosses. Genotypes are represented by large squares, which are divided into three regions, and chimaeras, comprising two different genotypes, are shown as pairs of squares joined by double-headed arrows. The *Gpi1* genotype is shown in the top half of each large square (e.g. *Gpi1^a/b^* is shown as a/b), the *Tyr* pigment genotype is shown in the bottom left small square (pigmented *Tyr^+/+^* is black and albino *Tyr^c/c^* is white) and the reiterated transgene genotype is shown, at the bottom right, as two circles (*Tg^+/+^* is two filled circles, *Tg^+/−^* is one filled and one empty circle and *Tg^−/−^* is two empty circles). (B) Diagram showing predicted outcomes of genetic crosses with chimaeras or their offspring. As in A, genotypes are represented by large squares but only the *Gpi1* genotype (top half of the squares) and *Tyr* pigment genotype (bottom half of the squares) are shown. Three *Tyr* pigment genotypes are illustrated: homozygous pigmented *Tyr^+/+^* (black rectangle), heterozygous pigmented *Tyr^+/c^* (black and white triangles) and albino *Tyr^c/c^* (white rectangle). The first part of diagram B shows that a cross between a *Gpi1^−/−^*↔*Gpi1^c/c^* null chimaera and an albino *Gpi1^c/c^* mouse may produce both pigmented and albino G1 generation offspring, but the frequency of pigmented offspring will vary, depending on the contribution of *Gpi1^−/−^* null cells to the germline of the chimaera. All pigmented, G1 generation offspring from *Gpi1^−/−^*↔*Gpi1^c/c^* chimaeras are expected to be *Gpi1^c/−^*. When these are crossed to *Tyr^+/+^*, *Gpi1^b/b^* mice, all the G2 generation offspring will be pigmented (either homozygous *Tyr^+/+^* or heterozygous *Tyr^+/c^*) and approximately 50% will be *Gpi1^b/−^* (identified as GPI1B phenotype by electrophoresis) and approximately 50% will be *Gpi1^b/c^* (GPI1BC phenotype). The second part of diagram B shows that a cross between other chimaeras, such as *Gpi1^a/−^*↔*Gpi1^c/c^*, and an albino *Gpi1^c/c^* mouse will produce pigmented, G1 offspring of two different *Gpi1* genotypes (*Gpi1^c/−^* and *Gpi1^a/c^* in the example shown). (C-L) Coats of the five female (C-G) and five male (H-L) *Gpi1^−/−^*↔*Gpi1^c/c^* chimaeras produced in the study. Chimaera reference numbers are shown with gender as F (female) or M (male). Chimaera 53F (panel G) was approximately 90% albino but the pigment patterns are not apparent in the photograph as the hair was sparse.

**Fig. 2.**
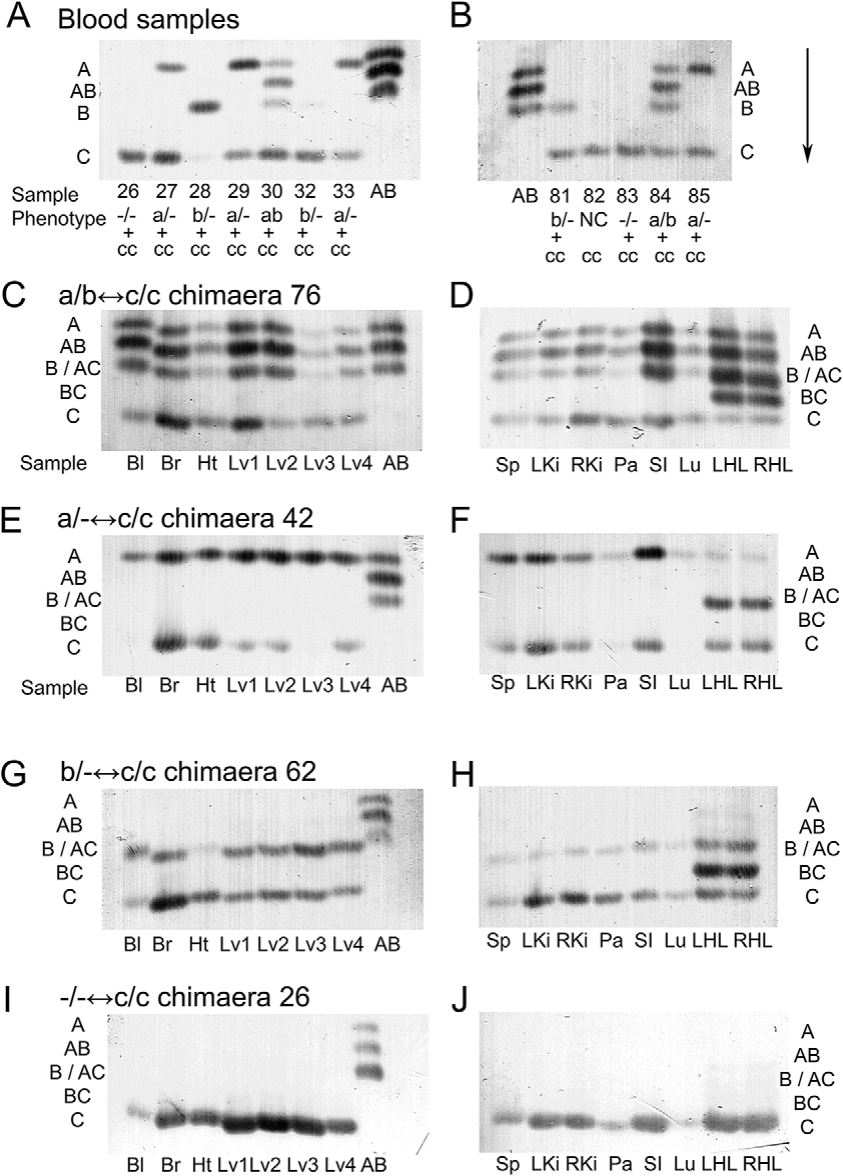
**Electrophoresis of GPI from tissues of different chimaeras.** (A,B) Blood samples. Chimaera reference numbers are shown under the lanes with the deduced sample genotypes (e.g. a/− + c/c=*Gpi1^a/−^*↔*Gpi1^c/c^* chimaera). NC is a non-chimaera (sample 82) and was distinguished from *Gpi1^−/−^*↔*Gpi1^c/c^* chimaeras by lack of coat and eye pigment. Sample AB is a blood sample from a *Gpi1^a/b^* heterozygote. Migration was in the direction of the arrow. (C-J) GPI electrophoresis of tissue samples from *Gpi1^a/b^*↔*Gpi1^c/c^* chimaera 76 (C,D), *Gpi1^a/−^*↔*Gpi1^c/c^* chimaera 42 (E,F), *Gpi1^b/−^*↔*Gpi1^c/c^* chimaera 62 (G,H) and *Gpi1^−/−^*↔*Gpi1^c/c^* chimaera 26 (I,J). Abbreviations of GPI allozyme bands: A, GPI1AA homodimer; AB, GPI1AB heterodimer; B, GPI1BB; B/AC, GPI1BB and/or GPI1AC; C, GPI1CC. Abbreviations of tissue samples: Bl, blood, Br, brain, Ht, heart. Lv1-Lv4, four liver samples (Lv1, medial lobe; Lv2, left lateral lobe, Lv3 right lateral lobe; Lv4, caudal lobe); Sp, spleen; LKi, left kidney; RKi, right kidney; Pa, pancreas; SI, small intestine; Lu, lung, LHL, left hind limb muscle; RHL, right hind limb muscle.

Ninety-two adult mice were produced by aggregation of embryos, produced by the genetic crosses summarised in Fig. 1A. Chimaeras were identified initially by their variegated coat and eye pigment, and the genotype combinations were deduced from their GPI electrophoresis phenotypes. Pigment and DNA *in situ* hybridisation to a reiterated *Tg* transgenic lineage marker were used as positive markers to identify the *Gpi1^−/−^* or *Gpi1^+/−^* cells in chimaeric tissues (Keighren et al., 2015). Sixty-seven mice were overt chimaeras with variegated coat pigmentation. Fifty-seven were classified as 16 *Gpi1^a/b^*↔*Gpi1^c/c^*, 26 *Gpi1^a/−^*↔*Gpi1^c/c^* and 15 *Gpi1^b/−^*↔*Gpi1^c/c^* chimaeras by GPI electrophoresis of blood samples taken at 1, 3 and 6-7.5 months (Fig. 2A,B). The remaining ten overt coat colour chimaeras (Fig. 1C-L) produced only GPI1C (e.g. chimaeras 26 and 83 in Fig. 2A,B) and these were provisionally classified as *Gpi1^−/−^*↔*Gpi1^c/c^* chimaeras. At this stage it remained possible that some were other genotype combinations with more than 97% GPI1C in the blood sample as less than 3% of one GPI1 allozyme may not always be detected (Kelly and West, 2002a). This was only likely for chimaeras 83 and 89 which both had only approximately 5% coat pigmentation and 3% Tg-positive nuclei in blood smears. However, all ten chimaeras were confirmed as being *Gpi1^−/−^*↔*Gpi1^c/c^* chimaeras by post-mortem analysis of other tissues (described below), breeding studies (chimaera 83) or both (chimaera 22). Although the frequency of overt chimaeras that were identified as *Gpi1^−/−^*↔*Gpi1^c/c^* chimaeras (10/67=14.9%) was lower than the expected frequency of 25%, this was not significant by a chi square goodness-of-fit test (*P*=0.078).

Of the remaining 25 mice that were not coat colour chimaeras, four were uniformly pigmented (1 *Gpi1^a/b^*, 1 *Gpi1^a/−^* and 2 *Gpi1^b/−^*) and 21 were entirely albino with only GPI1C detected in the blood. Some of these albino, GPI1C mice may have been cryptic chimaeras (including *Gpi1^−/−^*↔*Gpi1^c/c^* chimaeras) with a minor cell population that was excluded from coat and eye pigment and below detectable limits in the blood but this was not investigated further. Also, some may have been *Gpi1^−/−^*↔*Gpi1^c/c^* chimaeras, from which the *Gpi1^−/−^* cell population was eliminated by 1 month after birth.

### Test breeding to identify chimaeras with functional *Gpi1^−/−^* null germ cells

To investigate whether any of the ten *Gpi1^−/−^↔Gpi1^c/c^* chimaeras could produce functional gametes from homozygous *Gpi1^−/−^* null germ cells, they were crossed to albino *Gpi1^c/c^* mice, as shown in Fig. 1B. Chimaera 53 developed a tumour and was culled when it was pregnant with its first litter; all eight fetuses had unpigmented eyes and were GPI1C (expected genotype, *Gpi1^c/c^*). Seven other chimaeras each produced at least 45 first generation (G1) offspring, none of which was pigmented. At least three albino G1 offspring of each of these 7 chimaeras were typed for GPI1 and all were GPI1C (expected genotype, *Gpi1^c/c^*). Female chimaera 22 and male chimaera 83 produced both albino and pigmented offspring.

**Fig. 3.**
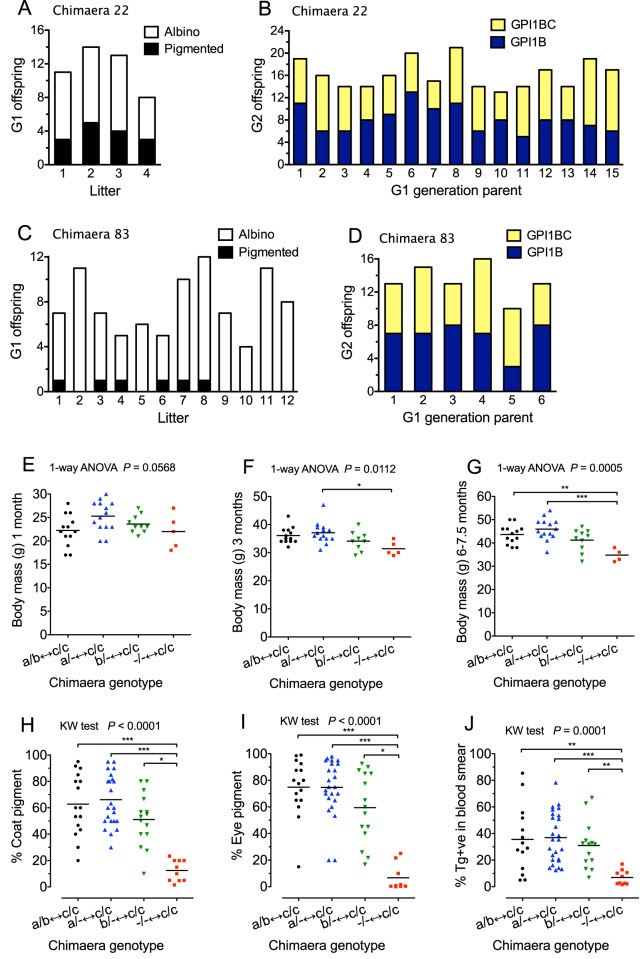
**Chimaera breeding experiments, body mass and composition.** (A-D) Results of breeding experiments with two putative *Gpi1^−/−^↔Gpi1^c/c^* chimaeras, showing production of some pigmented (putative *Gpi1^c/−^*), G1 generation offspring when crossed to albino, *Gpi1^c/c^* mice (A,C) and confirmation that all pigmented, G1 offspring produced both GPI1B (*Gpi1^b/−^*) and GPI1BC (*Gpi1^b/c^*) offspring in generation G2, when crossed to *Gpi1^b/b^* mice (B,D), as outlined in Fig. 1B. (E-G) Comparisons of body mass of male chimaeras of four genotypes at 1 month (E), 3 months (F) and 6-7.5 months (G). Genotypes were compared by one-way ANOVA, (*P*-values are shown on the graphs) and Tukey's multiple comparison test (asterisks). Females were not included as some were pregnant at 3 and 6-7.5 months. (H-J) Comparisons of composition of chimaeras of four genotypes from subjectively estimated percentage coat pigmentation (mean of estimates at 1, 3 and 6-7.5 months) (H), subjectively estimated percentage eye pigmentation (mean of left and right eyes) (I) and percentage of Tg-positive nuclei in blood smears at 3 months (J). Each point in the scatter plots represents the value for an individual chimaera. Genotypes were compared by Kruskal–Wallis test (*P*-values are shown) and Dunn's multiple comparison test (asterisks). Means are shown by horizontal bars. *P<0.05; **P<0.01; ***P<0.001.

Female chimaera 22 produced four litters with 15/46 (32.6%) pigmented offspring overall, as shown in Fig. 3A. All 15 pigmented G1 offspring and the 9 albino, G1 offspring that were tested (three from each of three litters) were GPI1C. Albino GPI1C, G1 offspring were expected to be *Gpi1^c/c^* homozygotes but pigmented GPI1C offspring were expected to be *Gpi1^c/−^* heterozygotes (Fig. 1B). The probability of all 15 pigmented G1 offspring being GPI1C if the chimaera was either *Gpi1^a/−^*↔*Gpi1^c/c^* or *Gpi1^b/−^*↔*Gpi1^c/c^* is only (1/2)^15^ (i.e. *P*=0.00003), which is very strong evidence that chimaera 22 was a *Gpi1^−/−^*↔*Gpi1^c/c^* chimaera. To check that all 15 pigmented G1 offspring were actually *Gpi1^c/−^* heterozygotes, they were crossed to pigmented, *Gpi1^b/b^* mice, as explained in Fig. 1B, and each produced at least two litters. The second generation (G2) offspring were all pigmented and were typed for GPI to check that all G1 offspring produced both GPI1B (*Gpi1^b/−^*) and GPI1BC (*Gpi1^b/c^*) G2 offspring and that approximately equal numbers were produced overall. In total, 122 G2 offspring were GPI1B and 120 were GPI1BC and Fig. 3B shows that all 15 G1 mice produced both GPI1B and GPI1BC G2 offspring. This implied that all the pigmented GPI1C G1 mice were *Gpi1^c/−^* rather than *Gpi1^c/c^*. The genetic crosses showed that chimaera 22 was a genuine *Gpi1^−/−^*↔*Gpi1^c/c^* chimaera with homozygous *Gpi1^−/−^* null germ cells that produced functional oocytes, which were capable of producing viable offspring when fertilised by wild-type spermatozoa. The composition of various body tissues in this chimaera is discussed below.

Male chimaera 83 produced 12 litters with 6/93 (6.5%) pigmented offspring overall (Fig. 3C). All six pigmented G1 offspring and the 18 albino G1 offspring tested (from six litters) were GPI1C. The probability of all 6 pigmented G1 offspring being GPI1C if the chimaera was either *Gpi1^a/−^*↔*Gpi1^c/c^* or *Gpi1^b/−^*↔*Gpi1^c/c^* is only (1/2)^6^ (i.e. *P*=0.0156), which is good evidence that chimaera 83 was a *Gpi1^−/−^*↔*Gpi1^c/c^* chimaera. The 6 pigmented G1 offspring were crossed to pigmented, *Gpi1^b/b^* mice and each produced at least two litters. In total, there were 40 GPI1B and 40 GPI1BC G2 offspring and all six G1 mice produced both GPI1B and GPI1BC G2 offspring (Fig. 3D). This confirms that the pigmented GPI1C G1 mice were all *Gpi1^c/−^* rather than *Gpi1^c/c^*. The genetic crosses imply that chimaera 83 was a genuine *Gpi1^−/−^*↔*Gpi1^c/c^* chimaera with homozygous *Gpi1^−/−^* null germ cells that produced functional spermatozoa, which were capable of fertilising wild-type oocytes and producing viable offspring. Unfortunately, this chimaera died at 6 months and tissues were not available for further analysis, so the genotype classification is based on the presence of a pigmented cell population, GPI electrophoresis of the blood and the absence of *Gpi1^a/c^* or *Gpi1^b/c^* mice among the six pigmented, G1 offspring. On this basis, male chimaera 83 was almost certainly a *Gpi1^−/−^*↔*Gpi1^c/c^* chimaera. However, as only blood was genotyped for GPI, this part of the study should be considered preliminary.

### Contribution of homozygous *Gpi1^−/−^* null cells to chimaeras

Chimaeras were killed at 6-7.5 months of age, after the test breeding was completed. Eyes were checked for pigment and a subjective estimate of the percentage of eye pigmentation was made for each eye for all the chimaeras. The initial genotype assignments, based on GPI electrophoresis of blood, were checked by GPI electrophoresis of the different body tissues and organs listed in the Materials and methods (Fig. 2C-J). Samples were analysed from nine *Gpi1^−/−^*↔*Gpi1^c/c^* chimaeras (including chimaera 53, which was culled before 6 months, but not chimaera 83, which died). All samples from all nine chimaeras only produced GPI1C bands, confirming that they were all *Gpi1^−/−^*↔*Gpi1^c/c^* chimaeras.

There was a trend for body mass of male *Gpi1^−/−^*↔*Gpi1^c/c^* chimaeras to be lighter than those in the other groups and for some comparisons this was significant at 3 and 6-7.5 months (Fig. 3E-G), suggesting that growth was affected. Female chimaeras were excluded from these comparisons as some were pregnant. Comparisons of subjective estimates of the percentage coat and eye pigmentation and the percentage of Tg-positive nucleated blood cells at 3 months all showed that *Gpi1^−/−^* null cells contributed much less than *Gpi1^a/b^*, *Gpi1^a/−^* or *Gpi1^b/−^* cells to chimaeras (Fig. 3H-J). This strongly suggests that, for these tissues at least, *Gpi1^−/−^* null cells were at a selective disadvantage, as previously reported for *Gpi1^−/−^*↔*Gpi1^c/c^* fetal chimaeras (Kelly and West, 2002a).

**Fig. 4.**
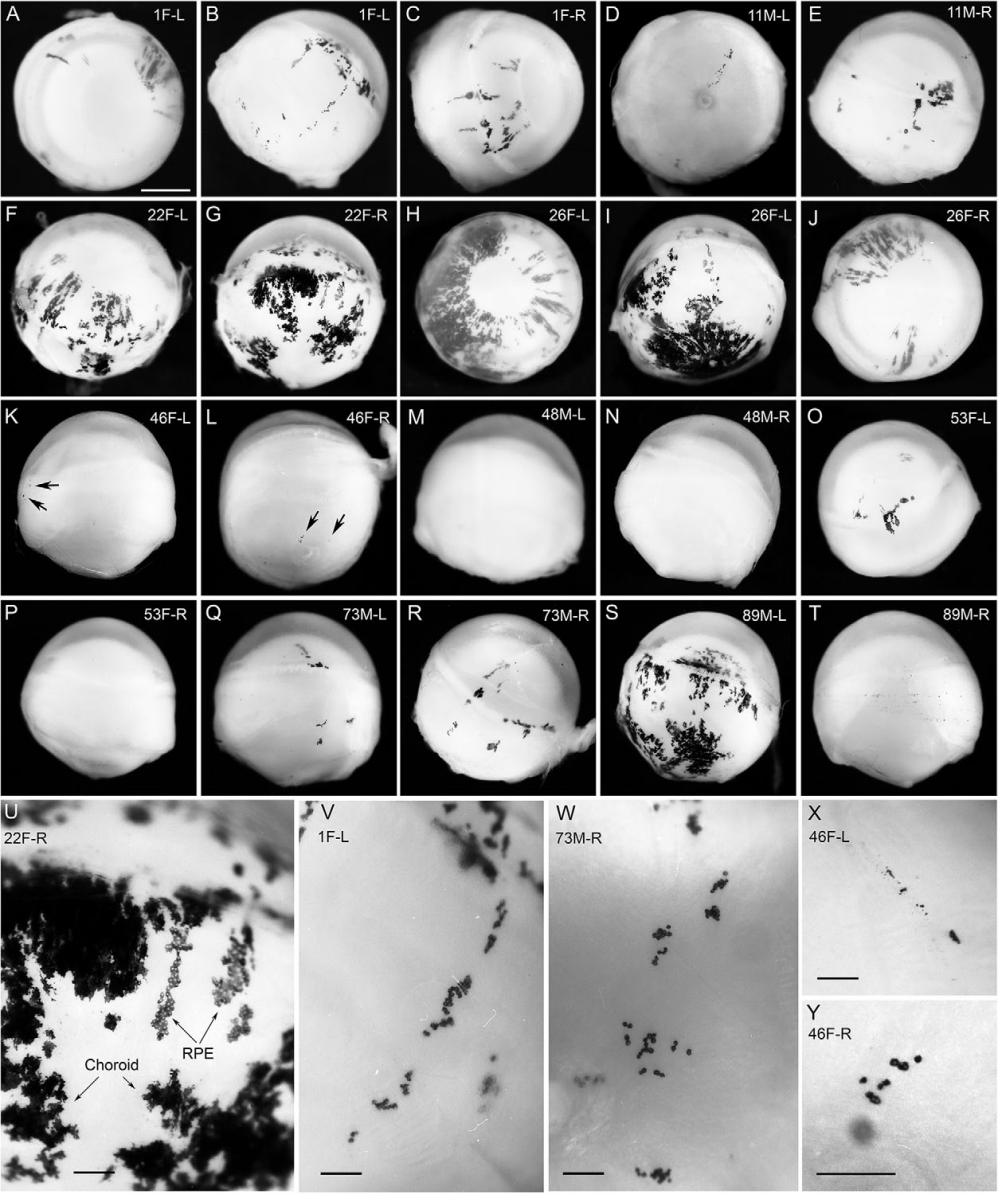
**Eye pigment in *Gpi1^−/−^***↔***Gpi1^c/c^* chimaeras.** (A-T) Whole eyes. Arrows in K and L indicate a few pigmented cells and pigmented regions in these eyes are shown at a higher magnification in X and Y. Eyes shown in M,N,P and T were unpigmented (small dark marks in T are not pigment). (U-Y) Higher power views showing pigmented regions of choroid (U,X) and underlying retinal pigment epithelium (U,V,W and Y). Chimaera reference numbers, gender (male, M or female, F) and left (L) or right (R) eyes are indicated. Scale bars: A-T, 1 mm (shown in A); U-Y, 200 µm.

Pigmented *Gpi1^−/−^* null cells tended to form radial stripes in the iris (Fig. 4H) and large patches in the choroid of *Gpi1^−/−^*↔*Gpi1^c/c^* chimaeric eyes (Fig. 4G,I,S,U), as reported for other chimaeras and mosaics (Gordon, 1977; West, 1976). Spatial distributions of pigmented *Gpi1^−/−^* null cells could also be seen in the retinal pigmented epithelium (RPE) of intact eyes of *Gpi1^−/−^*↔*Gpi1^c/c^* chimaeras if the overlying choroid was largely unpigmented (Fig. 4U-Y). Near the RPE periphery, some patches of pigmented RPE cells formed radial stripes in chimaera 22 (Fig. 4U), similar to those reported for other pigmented↔albino chimaeras and mosaics (Bodenstein and Sidman, 1987; Collinson et al., 2004; Hodson et al., 2011). In eyes of *Gpi1^−/−^*↔*Gpi1^c/c^* chimaeras with lower proportions of pigmented *Gpi1^−/−^* null cells, pigmented cells formed small clusters or discontinuous stripes in the RPE (Fig. 4V-Y).

**Fig. 5.**
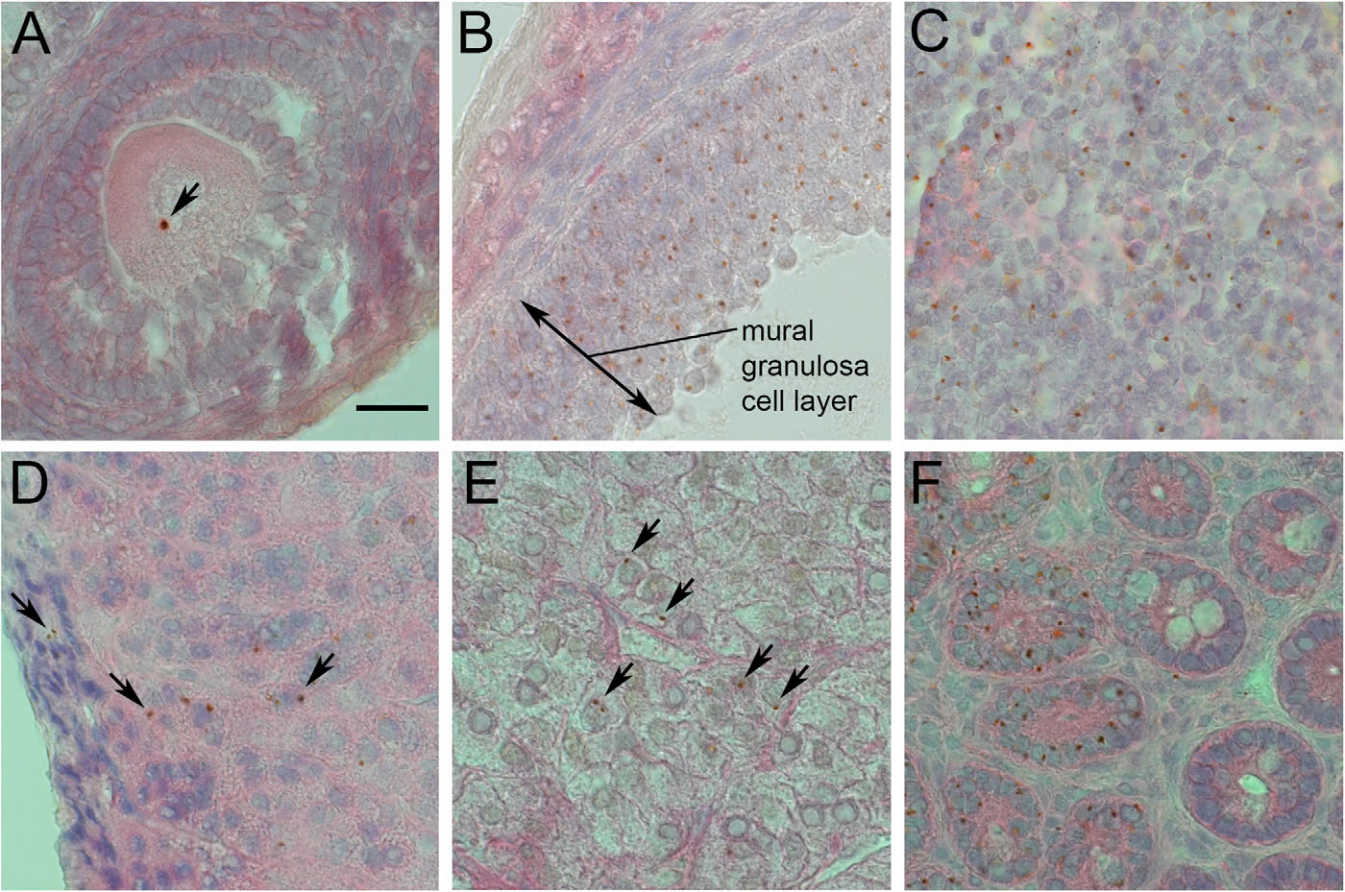
**Tg-positive *Gpi1^−/−^* null cells in different tissues of *Gpi1^−/−^***↔***Gpi1^c/c^* chimaera 22.** The Tg marker is identified as a small brown *in situ* signal (arrows) within some nuclei and the tissue sections are weakly stained with haematoxylin and eosin. The *in situ* signal is often not in the same plane of focus as the tissue section, so the cells sometimes appear out of focus. (A) Tg-positive oocyte in an ovarian pre-antral follicle. (B) Part of an ovarian antral follicle with abundant Tg-positive cells in the mural granulosa cell layer. (C) Region of thymus with abundant Tg-positive cells. (D) Adrenal cortex showing a single Tg-positive cell within the outer zona granulosa layer (top arrow) and a line of several Tg-positive cells in the zona fasciculata layer of the cortex (between other two arrows). (E) Adrenal medulla showing five Tg-positive cells (arrowed). (F) Cross section of small intestinal villi showing several villi with abundant Tg-positive cells on the left and other villi with no Tg-positive cells. Scale bar=20 µm.

DNA *in-situ* hybridisation (ISH) was used to detect the Tg lineage marker in several tissues of *Gpi1^−/−^*↔*Gpi1^c/c^* chimaeras and examples of tissues with Tg-positive cells are shown in Fig. 5 for female chimaera 22, which produced pigmented offspring in the genetic crosses. This includes a Tg-positive oocyte in a pre-antral ovarian follicle (Fig. 5A), which is consistent with the genetic evidence that chimaera 22 produced *Gpi1^−/−^* null oocytes. There were many Tg-positive mural granulosa cells in the part of the antral ovarian follicle shown in Fig. 5B but there were few Tg-positive cells elsewhere in the section. Tg-positive cells were abundant in some regions of sections of thymus (Fig. 5C) and spleen (not shown) from this particular chimaera but this was not studied quantitatively. In other tissues, Tg-positive cells tended to occur in isolation or in small clusters (Fig. 5D,E). The small group of Tg-positive cells in the adrenal cortex, shown in Fig. 5D, appeared to be radially aligned across the cortex as reported for other chimaeras and mosaics (Morley et al., 2004, 1996; Weinberg et al., 1985; West, 2001). This group of Tg-positive cells may have been produced by a Tg-positive, *Gpi1^−/−^* null stem cell as there is evidence that stem cells are located in the outer adrenal cortex and produce daughter cells that move inwards towards the medulla (Chang et al., 2013; King et al., 2009; Wood et al., 2013). In adult mice, the epithelium of intestinal villi is maintained by stem cells in the crypts and eventually each crypt harbours a single clone of stem cells (Ponder et al., 1985; Snippert et al., 2010). The section across several intestinal villi, in Fig. 5F, shows that epithelia of several villi are largely Tg-positive whereas others are Tg-negative, suggesting that Tg-positive, *Gpi1^−/−^* null stem cells are capable of maintaining intestinal villi.

**Fig. 6.**
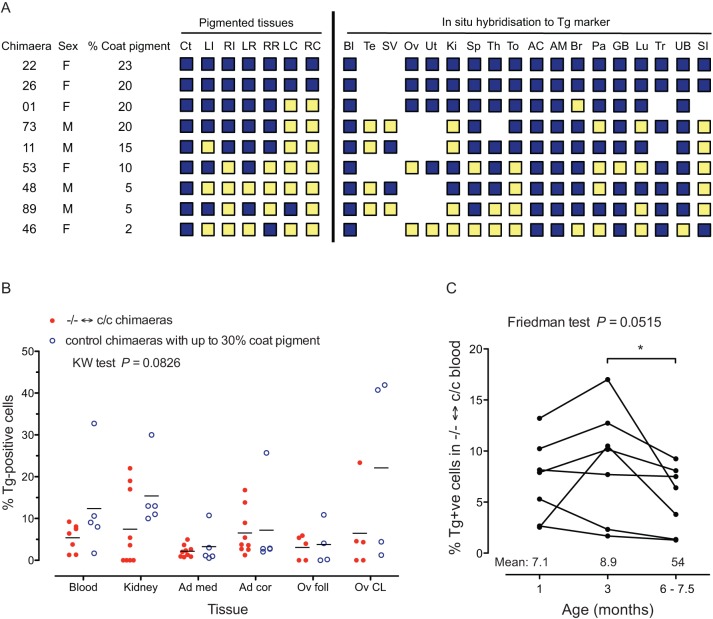
***Gpi1^−/−^* null cells in different tissues of *Gpi1^−/−^*↔*Gpi1^c/c^* chimaeras.** (A) Qualitative analysis of presence (dark blue square) or absence (yellow square) of *Gpi1^−/−^* null cells, detected as pigmented cells in seven tissues and as Tg-positive cells by *in situ* hybridisation in 18 unpigmented tissues. Abbreviations: AC, adrenal cortex; AM, adrenal medulla; Bl, blood smear; Br, brain; Ct, coat; F female; GB, gall bladder; Ki, kidney; LC, left choroid; LI, left iris; LR, left retinal pigment epithelium; Lu, lung; M, male; Ov, ovary; Pa, pancreas; RI, right iris; RC, right choroid; RR, right retinal pigment epithelium; SI, small intestine; SV, seminal vesicle; Sp, spleen; Te, testis; Th, thymus; To, tongue; Tr, trachea; UB, urinary bladder, Ut, uterus. (B) Quantitative comparisons of the percentage of Tg-positive cells identified in six tissues of seven *Gpi1^−/−^*↔*Gpi1^c/c^* chimaeras and five control chimaeras (*Gpi1^a/b^*↔*Gpi1^c/c^*, *Gpi1^a/−^*↔*Gpi1^c/c^* and *Gpi1^b/−^*↔*Gpi1^c/c^*) that were estimated to have no more than 30% coat pigment. Each point in the scatter plots represents the value for an individual chimaera. There were no significant differences between *Gpi1^−/−^*↔*Gpi1^c/c^* and control chimaeras for any tissue by Dunn's multiple comparison tests. Abbreviations: Ad cor, adrenal cortex; Ad med, adrenal medulla; Ov foll, ovarian follicle; Ov CL, ovarian corpora lutea. (C) Comparison of composition of the percentage of Tg-positive nuclei in blood smears taken at different ages from the same seven *Gpi1^−/−^*↔*Gpi1^c/c^* adult chimaeras. The mean values are shown at the bottom of the graph. The *P*-value for a Friedman test for repeated measures is shown on the graph and the significant *P*-values for Dunn's multiple comparison tests are shown by asterisks: *P<0.05.

*Gpi1^−/−^* null cells, identified either as pigmented or Tg-positive cells, were present in all the tissues and organ samples investigated in two females among the five female and four remaining male *Gpi1^−/−^*↔*Gpi1^c/c^* chimaeras analysed (Fig. 6A). However, we did not identify any Tg-positive cells in sections of testis that were examined from the four surviving male chimaeras. The other putative male *Gpi1^−/−^*↔*Gpi1^c/c^* chimaera was chimaera 83, which died before GPI genotyping of blood was confirmed with other tissues. If the preliminary genotype assignment for chimaera 83 is correct, the breeding results would imply that *Gpi1^−/−^* male germ cells could survive in the testis. We found no other organ from which *Gpi1^−/−^* null cells were consistently excluded. Although each of the nine *Gpi1^−/−^*↔*Gpi1^c/c^* chimaeras that were analysed had *Gpi1^−/−^* null cells in multiple tissues or organs, the frequency of samples with detectable *Gpi1^−/−^* null cells varied among chimaeras. The four *Gpi1^−/−^*↔*Gpi1^c/c^* chimaeras that were estimated to have at least 20% pigment in the coat had a significantly higher frequency of eye samples (RPE, choroid and iris in sections of left and right eyes) with pigment (20/24; 83%) than in the five chimaeras that were estimated to have less than 20% coat pigment (9/30; 30%; Fisher's exact test *P*=0.00011). Similarly, for the samples analysed by *in situ* hybridisation, there were significantly more samples with the Tg-marker in the chimaeras with at least 20% coat pigment (54/61; 89%) than in the chimaeras with less than 20% coat pigment (48/79; 61%; Fisher's exact test *P*=0.00024).

The percentages of Tg-positive nuclei were estimated from counts in blood smears and sections of kidneys, adrenal glands and ovaries for the nine surviving *Gpi1^−/−^*↔*Gpi1^c/c^* chimaeras and five control chimaeras (*Gpi1^a/b^*↔*Gpi1^c/c^*, *Gpi1^a/−^*↔*Gpi1^c/c^* or *Gpi1^b/−^*↔*Gpi1^c/c^*) that were judged to have no more than 30% coat pigment. Although the five control chimaeras were those with the lowest coat pigment they still had more coat pigment (mean=22.0%; range=10-30%) than those of the *Gpi1^−/−^*↔*Gpi1^c/c^* chimaeras (mean=13.3%; range=2-23%). The quantitative comparisons (Fig. 6B) showed that *Gpi1^−/−^* null cells made a very low contribution to these tissues in all the *Gpi1^−/−^*↔*Gpi1^c/c^* chimaeras and this was probably comparable to the lowest contributions of wild-type cells in control chimaeras. This provides further evidence for selection against homozygous *Gpi1^−/−^* null cells in *Gpi1^−/−^*↔*Gpi1^c/c^* chimaeras.

To test whether selection against *Gpi1^−/−^* null nucleated blood cells continued in adults, the percentages of Tg-positive *Gpi1^−/−^* null cells in blood smears taken at 1, 3 and 6-7.5 months were compared for the seven *Gpi1^−/−^*↔*Gpi1^c/c^* chimaeras that were analysed at each of these three time points (Fig. 6C). A Friedman test for repeated measures showed no overall differences among ages but Dunns multiple comparison tests indicated there was a reduction in the contribution of Tg-positive cells between 3 and 6-7.5 months. In principle, this could be attributable to selection against the *Gpi1^−/−^* null genotype, differences between the genetic backgrounds of the *Gpi1^−/−^* null cells and *Gpi1^c/c^* cells in the chimaera or stochastic variation. However, no such change occurred between 1 and 3 months so there is no convincing evidence for ongoing selection against homozygous *Gpi1^−/−^* null nucleated blood cells between 1 and 6-7.5 months.

Overall, it is clear that homozygous *Gpi1^−/−^* null cells make a very low contribution to all the tissues and organs of adult chimaeras that were tested and, in some individual samples, no *Gpi1^−/−^* null cells were identified. Although this suggests that *Gpi1^−/−^* null cells are at a general selective disadvantage there was no evidence that they were consistently excluded from specific tissues.

### Contribution of heterozygous *Gpi1^a/−^* and *Gpi1^b/−^* cells to chimaeras

**Fig. 7.**
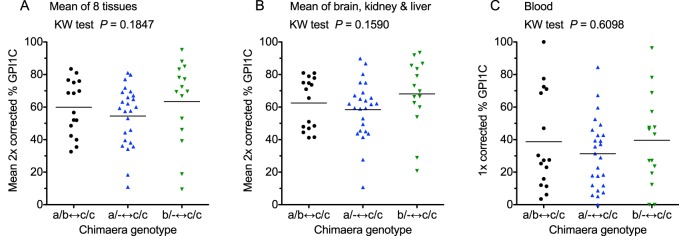
**Comparisons of compositions of three groups of chimaeras to test for selection against heterozygous *Gpi1^a/−^* and *Gpi1^b/−^* cells in chimaeras.** (A-C) Comparisons of corrected percentage GPI1C (estimate of percentage *Gpi1^c/c^* cells) in *Gpi1^a/b^*↔*Gpi1^c/c^*, *Gpi1^a/−^*↔*Gpi1^c/c^* and *Gpi1^b/−^*↔*Gpi1^c/c^* chimaeras from (A) mean composition of eight samples (brain, right kidney, medial liver lobe, heart, spleen, small intestine, pancreas and lung), (B) mean composition of three samples (brain, right kidney and medial liver lobe) and (C) composition of final blood sample (6-7.5 months). In A and B, the observed percentage GPI1C was corrected for reduced GPI1 production from *Gpi1^a/−^* and *Gpi1^b/−^* genotypes and for GPI1CC instability (2× corrected). In C, the observed percentage GPI1C was corrected for reduced GPI1 production from *Gpi1^a/−^* and *Gpi1^b/−^* genotypes but not for GPI1CC instability (1× corrected). Each point in the scatter plots represents the value for an individual chimaera. Means are shown by horizontal bars and genotypes were compared by Kruskal–Wallis test (*P*-values shown) and Dunn's multiple comparison test but there were no significant differences.

Although it was not possible to use quantitative GPI electrophoresis to investigate the contribution of *Gpi1^−/−^* null cells to *Gpi1^−/−^*↔*Gpi1^c/c^* chimaeras, it was possible to use this approach to determine whether *Gpi1^a/−^* or *Gpi1^b/−^* cells were at a selective disadvantage compared to *Gpi1^a/b^* cells in chimaeras by estimating the percentage GPI1C produced by *Gpi1^c/c^* cells in each chimaeric sample. Only eight of the tissue samples that were collected from each chimaera for GPI electrophoresis were quantified, as listed in the legend to Fig. 7. One complication is that the GPI1CC homodimer is less stable than GPI1AA, GPI1BB or GPI1AB. The observed percentage of GPI1C was corrected, both for the reduced stability of the GPI1CC homodimer and the reduced production of GPI1AA or GPI1BB by heterozygous *Gpi1^a/−^* or *Gpi1^b/−^* cells, as explained in the Materials and methods. Sex-specific samples were excluded to allow results for male and female chimaeras to be combined and skeletal muscle samples were excluded because they produced additional AC and/or BC heterodimers (Fig. 2D,F,H), which are difficult to quantify, as explained in the Materials and methods.

The corrected percentage GPI1C did not vary significantly among *Gpi1^a/b^*↔*Gpi1^c/c^*, *Gpi1^a/−^*↔*Gpi1^c/c^* and *Gpi1^b/−^*↔*Gpi1^c/c^* chimaeras for either the mean of all eight samples or the mean of three samples (brain, kidney and liver), that were selected to represent derivatives of the ectoderm mesoderm and endoderm respectively (Fig. 7A,B). As the percentage of GPI1C was not higher in *Gpi1^a/−^*↔*Gpi1^c/c^* or *Gpi1^b/−^*↔*Gpi1^c/c^* chimaeras than *Gpi1^a/b^*↔*Gpi1^c/c^* chimaeras, there was no evidence that heterozygous *Gpi1^a/−^* or *Gpi1^b/−^* cells were at a selective disadvantage compared to *Gpi1^a/b^* cells in chimaeras. Blood was not included in the samples analysed to produce a correction factor and GPI1CC may be less stable in blood than other tissues (Padua et al., 1978). As we had no specific correction factor for blood, the GPI1C band produced by blood in *Gpi1^a/b^*↔*Gpi1^c/c^*, *Gpi1^a/−^*↔*Gpi1^c/c^* and *Gpi1^b/−^*↔*Gpi1^c/c^* chimaeras was corrected for reduced GPI1 production from *Gpi1^a/−^* and *Gpi1^b/−^* genotypes but not for GPI1CC instability. For this reason, GPI results for blood were considered separately from the other eight tissues but this also provided no evidence for any selective disadvantage of *Gpi1^a/−^* or *Gpi1^b/−^* blood cells (Fig. 7C). Similarly, there was no evidence for selection against *Gpi1^a/−^* or *Gpi1^b/−^* cells from comparisons of pigmented tissues and Tg-positive cells in blood smears (Fig. 3H-J).

## DISCUSSION

### Contributions of homozygous *Gpi1*^−/−^ null cells to adult somatic tissues in chimaeras

The first aim of this study was to characterise the extent of survival of homozygous *Gpi1^−/−^* null cells in adult *Gpi1^−/−^*↔*Gpi1^c/c^* chimaeras. To label the *Gpi1^−/−^* null cells with a positive marker, we used the same pigment and reiterated transgenic (Tg) lineage markers that were used in earlier studies of *Gpi1^−/−^*↔*Gpi1^c/c^* chimaeras (Kelly and West, 2002a,b). Although the Tg marker is present in all nucleated cell types and hemizygous *Tg^+/−^* cells are developmentally neutral (Keighren et al., 2015), this is not an ideal marker as it is laborious to detect and not optimal for spatial analysis. Some fluorescent transgenic markers, driven by the endogenous *Rosa26* locus, (Ohtsuka et al., 2012) might be more suitable for future studies as, unlike some older reporter transgene markers, the newer markers appear to be expressed in all cell types and are not subject to mosaic expression. Nevertheless, the Tg marker was adequate to detect the presence of *Gpi1^−/−^* null cells in many tissues.

**Fig. 8.**
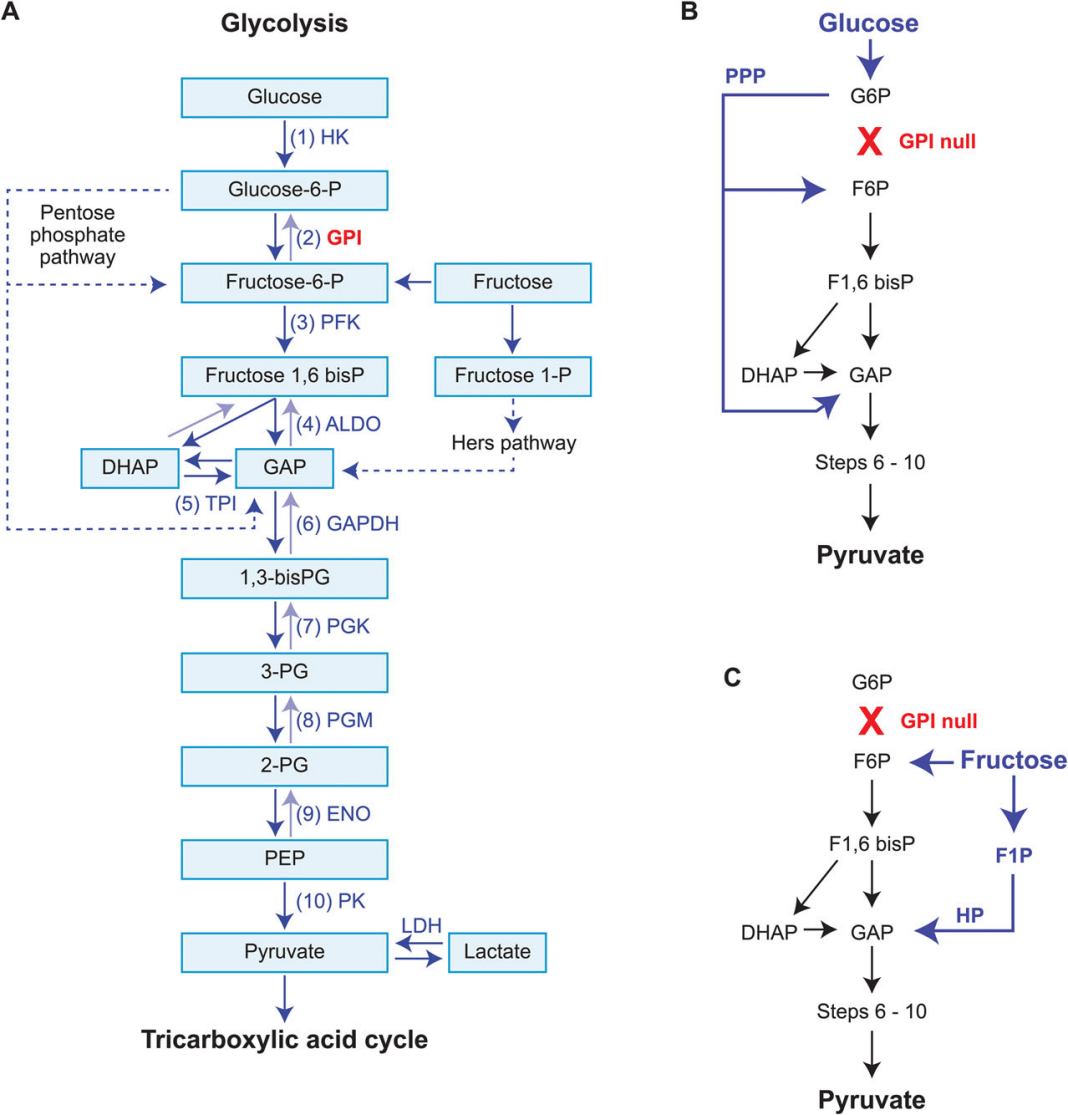
**Glycolysis.** (A) Outline of glycolysis showing relationship with both the pentose phosphate pathway and fructose, including the Hers pathway (dotted lines indicate where multiple steps are not shown). (B) Summary showing how the pentose phosphate pathway might, in principle, bypass a block in glycolysis at GPI by producing fructose 6-phosphate and glyceraldehyde 3-phosphate, which enter glycolysis at steps 3 and 6 respectively. However, this is likely to be inefficient. (C) Summary showing how fructose can act as a substrate for glycolysis and bypass a block in glycolysis at GPI, either if it is phosphorylated to fructose 6-phosphate, which enters glycolysis at step 3, or if its phosphorylated to fructose 1-phosphate, which enters the Hers pathway and produces glyceraldehyde 3-phosphate, which enters glycolysis at step 6. Seminal fluid is rich in fructose so fructose-fuelled glycolysis may explain how GPI1 null spermatozoa are able to function. If appropriate substrates for the TCA cycle are available, energy may also be produced in some cell types without involvement of glycolysis. The full enzyme names at each step in glycolysis are: (1) HK, hexokinase; (2) GPI, glucose phosphate isomerase; (3) PFK, phosphofructokinase; (4) ALDO, aldolase; (5) TPI, triosephosphate isomerase; (6) GAPDH, glyceraldehyde 3-phosphate dehydrogenase; (7) PGK, phosphoglycerate kinase; (8) PGM, phosphoglycerate mutase; (9) ENO, enolase; (10) PK, pyruvate kinase. Lactate dehydrogenase (LDH) is also shown. Other abbreviations: 1,3-bisPG, 1,3-bisphosphoglycerate; 2-PG, 2-phosphoglycerate; 3-PG, 3-phosphoglycerate; DHAP, dihydroxyacetone phosphate; Fructose-1-P (or F1P), fructose-1-phosphate; Fructose-1,6 bisP (or F1,6bisP), fructose 1,6-bisphosphate; Fructose-6-P (or F6P), fructose 6-phosphate; GAP, glyceraldehyde 3-phosphate; Glucose-6-P (or G6P), glucose 6-phosphate; HP, Hers pathway; PEP, phosphoenolpyruvate; PPP, pentose phosphate pathway.

Overall, the results showed that *Gpi1^−/−^* null cells usually made a very low contribution to *Gpi1^−/−^*↔*Gpi1^c/c^* chimaeras and were not detected in every tissue of all the chimaeras. Although homozygous *Gpi1^−/−^* null cells would be deficient in glycolysis, in many tissues they would be able to produce energy by the tricarboxylic acid (TCA) cycle and oxidative phosphorylation if appropriate substrates, such as lactate, pyruvate or glutamine, were available. The pentose phosphate pathway might also contribute to survival of *Gpi1^−/−^* null cells as, in principle, this pathway could by-pass the block in glycolysis at GPI (Fig. 8A,B). The pentose phosphate pathway begins with glucose-6-phosphate and, if it generates surplus ribose-5-phosphate, some is converted to fructose-6 phosphate and glyceraldehyde 3-phosphate. However, presumably neither the TCA cycle nor the pentose phosphate pathway can fully compensate for the GPI deficiency, otherwise *Gpi1^−/−^* null embryos would survive and *Gpi1^−/−^* null cells would not be severely depleted in chimaeras.

Some *Gpi1^−/−^* null cells in chimaeric tissues are probably rescued by neighbouring wild-type cells or blood, which could provide ATP or substrates for glycolysis downstream of GPI. The wild-type cells may not always need to be in the same tissue to support *Gpi1^−/−^* null cells. For example, the retinal pigment epithelium is a monolayer so *Gpi1^−/−^* null RPE cells could be supported by wild-type cells in the adjacent neural retina or choroid as well as the RPE itself. The survival of *Gpi1^−/−^* null nucleated blood cells (Tg-positive cells in blood smears) indicates that continuous, direct contact with neighbouring wild-type cells is not essential for survival of all *Gpi1^−/−^* null cells.

Previous evidence from fetal chimaeras showed that selection against *Gpi1^−/−^* null cells begins before E12.5 (Kelly and West, 2002a) but it is not known whether selection pressure decreases once there are only a small number of *Gpi1^−/−^* null cells that are scattered individually or in small groups among many more wild-type cells. Although no systematic spatial analysis was carried out, the distribution of *Gpi1^−/−^* null cells appeared to follow this pattern in some tissues. The depletion of *Gpi1^−/−^* null cells in chimaeras implies that they are at a selective disadvantage, at least during development, and that this glycolytic deficiency acts as a cell-autonomous defect. In both the present study and the earlier pilot study (Kelly and West, 2002b), a *Gpi1^−/−^*↔*Gpi1^c/c^* chimaera died of unknown causes before 6 months but it is not known whether this is just chance or if the presence of *Gpi1^−/−^* null cells affects the fitness of chimaeras.

### Production of functional oocytes from *Gpi1^−/−^* null germ cells in mouse chimaeras

In the breeding experiments, only 2/10 putative *Gpi1^−/−^*↔*Gpi1^c/c^* chimaeras (one female and one male) produced offspring from the pigmented, *Gpi1^−/−^* germ cell population. This is not surprising, however, because the albino, *Gpi1^c/c^* cell population predominated in all the *Gpi1^−/−^*↔*Gpi1^c/c^* chimaeras. Fifty percent of *Gpi1^−/−^*↔*Gpi1^c/c^* chimaeras are expected to be XX↔XY chimaeras, which are predicted to produce only albino offspring. This is because male XX↔XY chimaeras will only produce functional XY germ cells and female XX*↔*XY chimaeras will only produce functional XX germ cells (McLaren, 1975; Mullen and Whitten, 1971). In each case, these are expected to be genetically albino because the predominant somatic cell population of the developing gonad determines the sex of XX*↔*XY chimaeras. In these chimaeras the predominant cell population will be genetically albino and *Gpi1^c/c^*. The remaining 50% of *Gpi1^−/−^*↔*Gpi1^c/c^* chimaeras will be XX↔XX and XY↔XY chimaeras and could produce both pigmented and albino offspring. However, genetically albino, *Gpi1^c/c^* germ cells are likely to predominate so most offspring are expected to be albino.

Analysis with the Tg lineage marker showed that some *Gpi1^−/−^* null oocytes and ovarian follicle cells survived in several female *Gpi1^−/−^*↔*Gpi1^c/c^* chimaeras. Breeding experiments also showed that one of these adult female *Gpi1^−/−^*↔*Gpi1^c/c^* chimaeras produced offspring, which must have been generated by the fertilisation of *Gpi1^−/−^* null oocytes. Together with the previous preliminary report (Kelly and West, 2002b) this means that two adult female *Gpi1^−/−^*↔*Gpi1^c/c^* chimaeras have now been identified that produced functional *Gpi1^−/−^* null oocytes, which were fertilised and developed into fertile heterozygous *Gpi1^c/−^* offspring. Mouse oocytes produce energy by metabolising pyruvate via the TCA cycle whereas ovarian follicle cells mainly rely on glycolysis and can secrete pyruvate (Biggers et al., 1967; Boland et al., 1994; Donahue and Stern, 1968; Downs et al., 2002; Downs and Mastropolo, 1994; Downs and Utecht, 1999; Leese and Barton, 1985). Thus, wild-type follicle cells in chimaeric ovaries could rescue *Gpi1^−/−^* null oocytes by providing them with pyruvate or ATP and may also rescue neighbouring *Gpi1^−/−^* null follicle cells by providing them with ATP or intermediate glycolytic metabolites downstream of the GPI block at step 2 of glycolysis as discussed previously (Kelly and West, 2002b).

### Production of functional spermatozoa from *Gpi1^−/−^* null germ cells in mouse chimaeras

No adult male *Gpi1^−/−^*↔*Gpi1^c/c^* chimaeras had been identified previously, so one aim of the present study was to determine if male *Gpi1^−/−^*↔*Gpi1^c/c^* chimaeras had *Gpi1^−/−^* null germ cells that produced functional spermatozoa. The GPI composition of the blood of one male chimaera and the genetic breeding results implied that this was almost certainly a *Gpi1^−/−^*↔*Gpi1^c/c^* chimaera. However, this mouse died and other tissues were not available to confirm the GPI genotype assignment, so this provides only preliminary evidence that homozygous *Gpi1^−/−^* null germ cells can produce functional spermatozoa.

As we identified one male that was almost certainly a *Gpi1^−/−^*↔*Gpi1^c/c^* chimaera, we need to consider how homozygous *Gpi1^−/−^* null spermatogonia could survive in the chimaeric testis and generate functional, GPI-null spermatozoa that compete successfully, with wild-type spermatozoa in the female reproductive tract, to fertilise oocytes. Functional, haploid *Gpi1^−^* null spermatozoa are produced routinely by germ cells in heterozygous *Gpi1^+/−^* males. However, this is readily explained because the progeny of each A-type paired spermatogonia form a large syncytium of developing germ cells that are connected by cytoplasmic bridges (Greenbaum et al., 2011). The cytoplasmic bridges are large enough to allow exchange of cytoplasm, including mRNA, protein and even organelles (Ventela et al., 2003), so that genetically haploid sperm are considered to be phenotypically diploid (Braun et al., 1989). Survival of genetically haploid *Gpi1^−^* null spermatozoa produced by heterozygous *Gpi1^+/−^* males can, therefore, be explained because the spermatozoa will be phenotypically equivalent to diploid *Gpi1^+/−^* cells. In contrast, haploid *Gpi1^−^* null spermatozoa produced by homozygous *Gpi1^−/−^* null germ cells in a chimaera will be phenotypically equivalent to homozygous, diploid *Gpi1^−/−^* null cells because all the interconnected germ cells will be derived from the same homozygous *Gpi1^−/−^* null spermatogonium. Thus, we need to consider how the GPI block to glycolysis could be overcome by *Gpi1^−/−^* germ cells in the chimaeric testis and by phenotypically GPI-null, haploid *Gpi1^−^* spermatozoa in the female reproductive tract.

Sertoli cells support spermatogonia, spermatocytes and spermatids in the testis and generate lactate from glucose via glycolysis and lactate dehydrogenase (Jutte et al., 1983; Mita and Hall, 1982; Robinson and Fritz, 1981). Spermatocytes and round spermatids do not utilise glucose but convert lactate, secreted by the Sertoli cells, to pyruvate which produces energy via the TCA cycle and oxidative phosphorylation (Jutte et al., 1982, 1983; Mita and Hall, 1982; Nakamura et al., 1984, 1986). Thus, GPI-null spermatogonia, spermatocytes and spermatids should survive if they are supported by wild-type Sertoli cells, which will predominate in *Gpi1^−/−^↔Gpi1^c/c^* chimaeras. The survival of *Gpi1^−/−^* null somatic cells and germ cells in chimaeras may be wholly dependent on the presence of neighbouring wild-type cells. However, ejaculated spermatozoa, produced by male *Gpi1^−/−^* null germ cells, are not constantly in contact with wild-type cells, so their survival will depend on whether nutrients and alternative metabolic pathways are available to by-pass the block to glycolysis at GPI.

Glycolysis becomes important when spermatozoa reach the cauda epididymis, where they become motile, and glycolysis continues to be important after ejaculation (Miki, 2007). Both glycolysis and the TCA cycle are active in ejaculated spermatozoa but ATP production is compartmentalised. Enzymes that produce ATP by the TCA cycle are in the mitochondria of the mid-piece but enzymes that produce ATP by glycolysis are localised to the head and principal piece (du Plessis et al., 2015; Krisfalusi et al., 2006; Mukai and Travis, 2012; Westhoff and Kamp, 1997). Glucose is supplied to spermatozoa by the fluid of the female reproductive tract and from the spermatozoa's own glycogen stores (Ballester et al., 2000) and it is the substrate for both glycolysis and the pentose phosphate pathway, which generates NADPH. Seminal fluid is rich in fructose, which can replace glucose as the substrate for glycolysis (Fig. 8A,C) but cannot replace glucose in the pentose phosphate pathway (Fraser and Quinn, 1981; Goodson et al., 2012).

Different steps in fertilisation require ATP, generated by glycolysis or the TCA cycle, and NADPH, generated via the pentose phosphate pathway (Miki, 2007; Urner and Sakkas, 2003, 2005). Glycolysis is required for progressive motility of spermatozoa and, *in vitro* experiments show that motility can be supported by glycolysis, fuelled by glucose, fructose, mannose or sorbitol (Goodson et al., 2012). Capacitation of spermatozoa involves an increase in membrane fluidity, induction of hyperactivation and tyrosine phosphorylation of proteins (Naz and Rajesh, 2004; Visconti et al., 1995). This requires glycolysis and the pentose phosphate cycle (Aquila et al., 2010; Goodson et al., 2012; Miraglia et al., 2010) so it normally depends on glucose but tyrosine phosphorylation can occur *in vitro* if glucose is replaced by fructose (Goodson et al., 2012). The acrosome reaction requires lactate or pyruvate to drive the TCA cycle but does not require glucose (Miki, 2007; Urner and Sakkas, 1996) and sperm-oocyte fusion requires the pentose phosphate pathway (Urner and Sakkas, 2005).

GPI-null spermatozoa would be able to generate ATP by the TCA cycle but sperm motility may depend on local production of ATP by glycolysis in the principal piece (du Plessis et al., 2015; Westhoff and Kamp, 1997). GPI-null spermatozoa would also have an intact pentose phosphate pathway, so NADPH production should be unaffected (Fig. 8A,B). This pathway might also help by-pass the GPI block to glucose-fuelled glycolysis but fructose-fuelled glycolysis would probably be more effective. In principle, fructose might generate energy via glycolysis, after by-passing GPI, by one of two pathways (Fig. 8A,C). Although hexokinase has a lower affinity for fructose than for glucose, it can convert fructose to fructose-6-phosphate, which enters glycolysis, at step 3, having by-passed GPI. Alternatively, fructokinase can convert fructose to fructose-1-phosphate, which enters glycolysis at step 6 via the Hers pathway (Fraser and Quinn, 1981; Goodson et al., 2012; Hers, 1955).

Neither fructose-fuelled glycolysis nor the pentose phosphate pathway would by-pass blocks further down the glycolytic pathway. This is consistent with the infertility or sub-fertility of *Gapdhs^−/−^*, *Pgk2^−/−^* and *Eno4^Gt/Gt^* knockout male mice, which lack testis-specific forms of the glycolytic enzymes glyceraldeyhde-3-phosphate dehydrogenase, phosphoglycerate kinase and enolase, respectively (Danshina et al., 2010; Miki et al., 2004; Nakamura et al., 2013). In sperm, these enzymes are required for glycolysis steps 6, 7 and 9 (Fig. 8A). Male infertility or sub-fertility also occurs in *Ldhc*^−/−^ knockout mice that lack testis-specific lactase dehydrogenase enzyme, which interconverts pyruvate (the final product of glycolysis) and lactate (Odet et al., 2008, 2011). Thus, if fructose acted as a substrate for glycolysis, this should bypass the GPI block at step 2 of glycolysis in phenotypically GPI-null, haploid *Gpi1^−^* null spermatozoa that are produced by homozygous *Gpi1^−/−^* null germ cells.

### Conclusions

Chimaera analysis proved to be an inefficient approach to study the fate of *Gpi1^−/−^* null germ cells as only two of ten *Gpi1^−/−^*↔*Gpi1^c/c^* chimaeras produced offspring from the genetically pigmented, *Gpi1^−/−^* cell population. A more detailed investigation of the rescue of *Gpi1^−/−^* null, male germ cells and the phenotypically GPI1 null spermatozoa that they produce requires other approaches. For example, *Gpi1* could be conditionally knocked out, specifically in male germ cells using Cre-*loxP* transgenic mice with a spermatocyte-specific or spermatid-specific Cre-driver (Smith, 2011). Nevertheless, our experiments with adult *Gpi1^−/−^↔Gpi1^c/c^* chimaeras showed that, although *Gpi1^−/−^* null cells are at a selective disadvantage, some could survive in adult somatic tissues. These are likely to be rescued by neighbouring wild-type cells. Genetic breeding experiments supported the previous, preliminary report that *Gpi1^−/−^* null oocytes can survive and be fertilised and they also provided preliminary evidence that homozygous *Gpi1^−/−^* null germ cells can produce functional spermatozoa. Wild-type follicle cells are thought to support *Gpi1^−/−^* oocytes and wild-type Sertoli cells are likely to support *Gpi1^−/−^* spermatogonia, spermatocytes and spermatids. We suggest that, for phenotypically GPI-null spermatozoa, the deficiency in glucose-fuelled glycolysis may be by-passed by fructose-fuelled glycolysis. This is only feasible because GPI is an early step in glycolysis and defects in enzymes required for later steps of glycolysis would not be rescued in this way.

## MATERIALS AND METHODS

### Mice

All work with mice (*Mus musculus* Linnaeus) was performed in accordance with institutional guidelines and UK Home Office regulations (licences PPL 60/1150 and PPL 60/1989). Mice were house under conventional conditions in the University of Edinburgh, Medical School. Two pigmented (*Tyr^+/+^*) stocks (designated ‘GN’ and ‘NUL’), carrying the *Gpi1^a−m1H^* (*Gpi1^−^*) null allele (Pearce et al., 1995; Peters and Ball, 1990), were maintained as *Gpi1^a/−^* and *Gpi1^b/−^* genotypes by crossing *Gpi1^a/−^* to *Gpi1^b/b^* and *Gpi1^b/−^* to *Gpi1^a/a^* mice in alternate generations as previously described (Kelly and West, 2002a,b). Stock ‘NUL’ was homozygous (*Tg^+/+^*) for the reiterated β-globin transgene lineage marker *TgN(Hbb-b1)83Clo* (Katsumata and Lo, 1988; Lo, 1986; Lo et al., 1987), which we used as a target for DNA *in situ* hybridisation (Keighren and West, 1993). The abbreviation *Tg^−/−^* denotes mice without the reiterated transgene, *Tg^+/−^* denotes hemizygotes and *Tg^+/+^* denotes homozygotes.

### Chimaera production

Adult mouse chimaeras (series AdCK) were produced by aggregating pairs of preimplantation embryos (Tarkowski, 1961), as described elsewhere (Kelly and West, 2002a). The genetic crosses used to produce chimaeras are summarised in Fig. 1A. Genetically pigmented (*Tyr^+/+^*), 8-cell stage, *Tg^+/−^* embryos of four *Gpi1* genotypes (*Gpi1^a/b^*, *Gpi1^a/−^*, *Gpi1^b/−^* and *Gpi1^−/−^*) were produced by crossing *Tyr^+/+^*, *Gpi1^b/−^*, *Tg^−/−^* GN mice to *Tyr^+/+^*, *Gpi1^a/−^*, *Tg^+/+^* NUL mice. Embryos were flushed from the reproductive tract of mated superovulated females at E2.5 and aggregated with 8-cell stage, genetically albino (*Tyr^c/c^*), *Gpi1^c/c^* embryos, without the *Tg* transgene (designated ‘CF_2_’ embryos). These CF_2_ embryos were produced by intercrossing (C57BL-*Gpi1^c^,Tyr^c^*/Ws×BALB/c-*Gpi1^c^*/Ws)F_1_ hybrids (abbreviated to ‘CF_1_’ hybrids). After overnight culture, the E3.5 chimaeric aggregates were transferred to the uteri of pseudopregnant homozygous *Gpi1^c/c^*, ‘CF_1_’ hybrid females 2.5 days after mating to vasectomised males and allowed to go to term. This resulted in pigmented *Tg^+/+^*↔albino *Tg^−/−^* chimaeras of four *Gpi1* genotype combinations: *Gpi1^a/b^*↔*Gpi1^c/c^*, *Gpi1^a/−^*↔*Gpi1^c/c^*, *Gpi1^b/−^*↔*Gpi1^c/c^* and *Gpi1^−/−^*↔*Gpi1^c/c^*.

### Analysis of chimaeras

Putative chimaeras were weighed and the percentage coat pigmentation was estimated subjectively at 1, 3 and 6-7.5 months, when they were killed by cervical dislocation. Blood samples were taken from the tail vein of anaesthetised live mice at 1 and 3 months. Immediately after mice were killed at 6-7.5 months, a final blood sample was taken and various tissues and organs were collected, rinsed in PBS and blotted dry. Eyes were examined under a dissecting microscope and the overall percentage eye pigmentation was estimated subjectively. Blood smears and samples of some solid tissues and organs were prepared for histology and DNA *in situ* hybridisation as described below. Other samples were homogenised in distilled water with a Polytron homogeniser and stored at −20°C in 1.5 ml microtubes for GPI electrophoresis. Samples analysed by GPI electrophoresis included blood, brain, heart, spleen, left and right kidneys, four liver lobes (medial, left lateral, right lateral and caudal), pancreas, small intestine (duodenum), lung, muscles from all four limbs, tongue and either seminal vesicle or the right uterine horn.

### GPI electrophoresis

Cellulose acetate electrophoresis and staining for GPI activity was carried out as described previously (West and Flockhart, 1994) to separate the GPI1C allozyme band (GPI1CC homodimer), encoded by the *Gpi1^c^* allele, from GPI1A, GPI1AB and GPI1B allozyme bands, encoded by the *Gpi1^a^* and *Gpi1^b^* alleles. Blood samples were used for initial genotype assignments and this was checked by electrophoresis of a range of tissue samples collected post-mortem. Images of the stained electrophoresis plates were obtained using a flatbed scanner (Epson V330 photo), cropped using Adobe Photoshop CS6 software (Adobe Systems Inc. San Jose, CA) and converted to high-contrast, greyscale images using the auto contrast function.

To estimate the percentage contribution of *Gpi1^c/c^* cells in *Gpi1^a/b^*↔*Gpi1^c/c^*, *Gpi1^a/−^*↔*Gpi1^c/c^* and *Gpi1^b/−^*↔*Gpi1^c/c^* chimaeras, the percentage GPIC band was estimated in a selected group of tissues and organs by scanning densitometry (West and Flockhart, 1994). However, the GPI1CC homodimer is less heat stable than GPI1AA, GPI1BB and GPI1AB dimers (Padua et al., 1978). A correction factor, for the reduced stability of GPI1CC, was derived by estimating the relative activity of different GPI bands after electrophoresis of three series of 1:1 mixtures (by weight) of tissue homogenates from *Gpi1^a/b^*, *Gpi1^a/−^*, *Gpi1^b/−^* and *Gpi1^c/c^* mice. Each of three mixtures (*Gpi1^a/b^*+*Gpi1^c/c^*, *Gpi1^a/−^*+*Gpi1^c/c^* and *Gpi1^b/−^*+*Gpi1^c/c^* mixtures) was prepared three times (using different mice each time) for eight samples (brain, right kidney, medial liver lobe, heart, spleen, small intestine, pancreas and lung).

Although most tissues of chimaeras show an additive banding pattern of the two constituent genotypes, skeletal muscle samples (including tongue) produce additional bands. This is because muscle development involves fusion of myoblasts (Mintz and Baker, 1967) and GPI is a dimer. If myoblasts with different *Gpi1* genotypes fuse in chimaeras, two or more types of monomer are produced in the multi-nucleated muscle fibres and all dimer combinations can occur. For example, most tissues of *Gpi1^a/b^↔Gpi1^c/c^* chimaeras produced the *Gpi1^a/b^* banding pattern (A, AB and B bands, representing AA, AB and BB dimers) plus the *Gpi1^c/c^* banding pattern (C bands comprising CC homodimers) but skeletal muscle also produced AC and BC heterodimer bands. Skeletal muscle samples were excluded from the quantitative analysis both because the GPI1AC band co-migrated with GPI1B and because the relative activities of the GPI1AC and GPI1BC heterodimer bands were not determined so correction factors were not calculated.

The mean percentage GPI1C (±95% confidence interval) for the eight sample mixtures was 34.62±3.04% for mixtures of *Gpi1^a/b^* and *Gpi1^c/c^*, 51.72±4.03% for mixtures of *Gpi1^a/−^* and *Gpi1^c/c^* and 53.87±4.58% for mixtures of *Gpi1^b/−^* and *Gpi1^c/c^*. As GPI1C values did not differ significantly among tissues and organs by one-way analysis of variance (ANOVA) the same correction factor was used for each of the eight samples. For the 1:1 *Gpi1^a/b^* *+* *Gpi1^c/c^* mixtures, the (AA+AB+BB)/CC band ratio (R) was greater than 1.0 (65.38/34.62=1.89), implying that *Gpi1^c/c^* cells had less GPI activity than *Gpi1^a/b^* cells. For *Gpi1^a/−^* *+* *Gpi1^c/c^* and *Gpi1^b/−^* *+* *Gpi1^c/c^* mixtures, the corresponding band ratios were respectively 0.93 (48.28/51.72) for AA/CC and 0.86 (46.13/53.87) for BB/CC. These were lower than for *Gpi1^a/b^* *+* *Gpi1^c/c^* mixtures because *Gpi1^a/−^* and *Gpi1^b/−^* hemizygotes produce only about half as much GPI1 activity as *Gpi1^a/b^* heterozygotes. The band ratios (R) were used to correct the observed percentage GPI1C (C_o_) for both the greater instability of GPI1CC and the reduced GPI1 production from *Gpi1^a/−^* and *Gpi1^b/−^* genotypes, such that the corrected percentage GPI1CC band=C_o_×R×100/[(C_o_×R)+(100−C_o_)].

### Histology and DNA *in situ* hybridisation

Blood smears were air dried on clean microscope slides, fixed in acetic alcohol (3 ethanol:1 acetic acid, v/v) for 60 min, air dried, immersed in acetone for 10 min and dehydrated through graded alcohols before *in situ* hybridisation (ISH). Tissue samples for DNA ISH or eye histology for pigment analysis were fixed in acetic alcohol. After fixation, lenses were removed from the eyes, through a cut made in the cornea, to facilitate sectioning. Samples of solid tissues were processed to paraffin wax for histology. Sections were cut at 7 µm thickness and mounted on glass microscope slides coated with 3-aminopropyltriethoxysilane (TESPA; Sigma-Aldrich, Poole, UK). Tissue sections and blood smears were analysed by DNA ISH to the transgene and hybridised digoxygenin-labelled DNA probe was detected by diaminobenzidine (DAB) staining for peroxidase-labelled antibody as described previously (Keighren and West, 1993). Slides were counterstained with haematoxylin and eosin and examined by bright-field microscopy to identify *Tg*^+/−^ cells derived from the *Gpi1^a/b^, Gpi1^a/−^*, *Gpi1^b/−^* or *Gpi1^−/−^* (GN×NUL) component in the chimaeras by the presence of a brown hybridisation signal in the nucleus.

For most tissues in the chimaeras, the contribution of Tg-positive cells was scored qualitatively as positive or negative for the presence of nuclei containing the hybridisation signal after ISH. Tissue sections with Tg-positive nuclei were photographed using a calibrated Zeiss Axiovision 4.8 digital camera system on a Zeiss Axioplan 2 compound microscope. Quantitative counts of Tg-positive nuclei were made for blood smears and sections of kidneys, adrenals and ovaries using a Leica Diaplan compound microscope with a 10×10 eyepiece grid. Crude counts of Tg-positive nuclei were corrected using the percentage of Tg-positive nuclei seen in the equivalent tissues from hemizygous *Tg^+/−^* positive control mice. For each tissue section or blood smear, approximately 300 nuclei were scored for the presence of the hybridisation signal. *Tg^−/−^* negative control sections were also included as quality controls in each *in situ* hybridisation run.

### Test breeding to evaluate gamete function

To test whether putative *Gpi1^−/−^*↔*Gpi1^c/c^* chimaeras contained a *Gpi1^−/−^* null germ cell population that could produce functional gametes, *Gpi1^−/−^*↔*Gpi1^c/c^* chimaeras were crossed to albino *Gpi1^c/c^* mice, as outlined in Fig. 1B. At least three albino first generation (G1) offspring of each chimaera and all pigmented G1 offspring were typed for GPI to check they were all GPI1C. Albino GPI1C, G1 offspring were expected to be *Gpi1^c/c^* homozygotes whereas pigmented GPI1C, G1 offspring were expected to be *Gpi1^c/−^* heterozygotes. To check that all pigmented G1 mice were *Gpi1^c/−^* heterozygotes, they were crossed to *Gpi1^b/b^* mice to produce G2 offspring. G2 mice were typed for GPI1 to check there were approximately equal numbers of GPI1B (*Gpi1^b/−^*) and GPI1BC (*Gpi1^b/c^*) individuals (Fig. 1B).

### Statistics

The choice of parametric or non-parametric tests was guided, in part, by normality tests. GraphPad Prism 5.0c (GraphPad Software, Inc. San Diego, CA) was used for most statistical tests, as described in the text. An online statistical calculator (http://vassarstats.net/index.html) was used for chi square goodness-of-fit tests.
